# Archaeal Tubulin-like Proteins Modify Cell Shape in *Haloferax volcanii* during Early Biofilm Development

**DOI:** 10.3390/genes14101861

**Published:** 2023-09-25

**Authors:** Alexei Cooper, Andrea M. Makkay, R. Thane Papke

**Affiliations:** Department of Molecular and Cell Biology, University of Connecticut, Storrs, CT 06268, USA; alexei.cooper@uconn.edu (A.C.); makkay@uchc.edu (A.M.M.)

**Keywords:** *Haloferax volcanii*, tubulin, CetZ, FtsZ, morphology, biofilm, archaea

## Abstract

Tubulin, an extensively studied self-assembling protein, forms filaments in eukaryotic cells that affect cell shape, among other functions. The model archaeon *Haloferax volcanii* uses two tubulin-like proteins (FtsZ1/FtsZ2) for cell division, similar to bacteria, but has an additional six related tubulins called CetZ. One of them, CetZ1, was shown to play a role in cell shape. Typically, discoid and rod shapes are observed in planktonic growth, but under biofilm formation conditions (i.e., attached to a substratum), *H. volcanii* can grow filamentously. Here, we show that the deletion mutants of all eight tubulin-like genes significantly impacted morphology when cells were allowed to form a biofilm. *ΔftsZ1*, *ΔcetZ2*, and *ΔcetZ4-6* created longer, less round cells than the parental and a higher percentage of filaments. *ΔcetZ1* and *ΔcetZ3* were significantly rounder than the parental, and *ΔftsZ2* generated larger, flat, amorphic cells. The results show all tubulin homologs affect morphology at most timepoints, which therefore suggests these genes indeed have a function.

## 1. Introduction

Tubulin is an extensively studied self-assembling protein that uses its GTPase function to form filaments that affect cell shape but also performs several other important functions in eukaryotic cell biology, including segregating chromosomes during cell division and working with actin to form the structural cytoskeleton [1]. Neither actin nor tubulin is directly found in prokaryotes; however, most bacteria and some archaea have distantly related homologs [2]. In bacteria, the tubulin-like homolog *ftsZ* occurs singularly in chromosomes, and the proteins self-assemble during the cell division cycle to create a “Z ring” that constricts to separate the two daughter cells [3,4]. They are not involved in maintaining cell shape, and deletions cause cells to grow poorly or not at all [3]. In addition to FtsZ, many archaeal species have another family of tubulin-like homologs called CetZ (previously considered a third FtsZ group [5]), which, unlike FtsZ, have not been well studied, and therefore, little is known about them beyond their structure and probable GTPase ability [5,6]. Distribution analyses have shown that CetZ homologs are patchily distributed, the number of homologs differs from species to species, and they are only found in archaeal species that also encode FtsZ [6].

The model archaeon, *Haloferax volcanii*, has eight tubulin-like homologs comprised of two FtsZs and six CetZ’s [7]. Both FtsZ proteins self-assemble and are used in conjunction during cell division to form the Z-ring structure. FtsZ1 acts as a scaffold at the midcell for FtsZ2, which then helps constrict and divide the cell [8,9]. Unlike bacteria that require FtsZ, *H. volcanii* cells are viable with minimal growth deficiencies when both *ftsZ1* and *ftsZ2* are deleted, suggesting alternative propagation mechanisms [9]. Though *H. volcanii* has six CetZ proteins, other haloarchaea like *Haloferax mediterranei* and *Halobacterium salinarum* only have four and three, respectively. Many other archaea, like methanomicrobia, thermococci, and archaeoglobi, also have CetZ proteins [6], though studies have only focused on *H. volcanii*. Though some members of the TACK (Thaumarchaeota, Aigarchaeota, Crenarchaeota, Korarchaeota) and Asgard archaeal groups have FtsZ, so far, no CetZ proteins have been found [6]. CetZ proteins in *H. volcanii* are not required for cell division, and only CetZ1 is implicated as a requirement for rod-shape development in shaking liquid cultures [7]. Under standard growth conditions (i.e., shaking and planktonic), none of the other CetZ proteins were reported to have an observable phenotype, perhaps suggesting functional redundancy in the remaining five. However, in a study on *H. volcanii* biofilms (biofilms are defined as cells attached to a surface, encased in an exopolymeric matrix [10,11,12], and phenotypically different from planktonic cells [13,14]), cells exhibited morphological changes going from circular and flat (disk-shaped) while shaking to two co-existing forms when they became sessile: a rod and a filamentous shape [15]. While the rod shape has been observed previously in shaking cultures, the filamentous morphology appears only under biofilm conditions in *H. volcanii* [15], suggesting different tubulin homologs might function to alter cell shape under different biological conditions.

The archaeal tubulin-like protein family has shown its significance in a limited number of non-tubulin-focused studies. For example, a transcriptome analysis performed on *H. volcanii* identified differentially expressed genes during the formation of biofilms (conditions that facilitate horizontal gene transfer (HGT) [16,17]). Transcriptome analyses of planktonic cells and cells initiating a biofilm (during the first eight hours) showed that all eight tubulin-like genes were differentially expressed within at least two hours of becoming attached [18]. Interestingly, six of the genes had increased expression, whereas *cetZ2* and *cetZ3* had decreased expression. In a different study, FtsZ1, FtsZ2, CetZ1, and CetZ5 co-precipitated with LonB, an archaeal LonA homolog [19]. In *E. coli*, LonA is a protease that helps release FtsZ inhibition by degrading its inhibitor, SulA, towards the end of the SOS response [19]. Differences in co-precipitation among the tubulin family homologs in *H. volcanii* could suggest separate or different functions.

With the exception of CetZ1, no function has been determined for any of the remaining five CetZ homologs. CetZ2 has been implicated in cell shape only when overexpressed; deletion of CetZ2 showed no impact on cell shape. Additionally, none of the tubulin-like proteins (FtsZs/CetZs) have been analyzed for their effect on cell shape during biofilm formation. However, it is clear that they are potentially involved in several processes, indicating their biological importance is under-evaluated and in need of further investigation. Given the observations that, under biofilm growth conditions, *H. volcanii* cells change their morphology; archaeal tubulin-like homolog CetZ1 determines shape under shaking conditions; five tubulin-like homologs have an unknown function; and all tubulin-like homologs were differentially expressed during biofilm development or precipitated alongside LonB, we decided to analyze all eight tubulin family genes for function in determining cell shape under biofilm development conditions and report our results here.

## 2. Materials and Methods

### 2.1. Culture Methods

All *Haloferax volcanii* strains used (Table S1) have a H53 (*∆pyrE2*, *∆trpA*) background, which is auxotrophic for uracil and tryptophan. Cultures were grown in casamino acid (Hv-CA) medium at 42 °C with 160 rpm shaking. Hv-CA medium contained 144 g of NaCl, 21 g of MgSO_4_·7H_2_O, 18 g of MgCl_2_·6H_2_O, 4.2 g of KCl, 12 mM of Tris HCl (pH 7.5), and 5.0 g of casamino acids per liter [20]. A total of 2% agar (*w*/*v*) was added for solid media. When needed, 50 µg/mL uracil, 50 µg/mL tryptophan, and/or 5-fluoroorotic acid (50 μg/mL) were supplemented. Trace element solution [16] was added to all liquid media for biofilm growth. All cells required tryptophan to be supplemented for growth, but strains expressing the pNUFsmRS-GFP plasmid for microscopy visualization did not require uracil.

*Escherichia coli* strains were grown at 37 °C while shaking at 200 rpm in S.O.C. (Clontech, Cat. #636763) or LB media with ampicillin (100 μg/mL) supplemented as needed. LB medium contained 5 g of NaCl, 5 g of tryptone, and 2.5 g of yeast extract per 500 mL; solid media required 1.5% agar (*w*/*v*). For white–blue screening, 40 μL of X-gal (20 mg/mL) was spread onto plates when needed.

### 2.2. Strain Construction

To construct deletion mutants, flanking regions of the intended gene 800–1200 bp in length were PCR-amplified (for primer list, see Table S1) with a 15 bp linker included to allow for the upstream and downstream fragments to recombine [21]. The flanking regions were cloned into linearized plasmid pTA131 using the In-Fusion HD Cloning Kit (Clontech, San Jose, CA, USA) and transformed into Stellar-competent cells (Clontech, Cat. #636766). Using LB-Amp plates with X-gal, white colonies were screened for successful plasmid assembly by PCR. Properly assembled deletion plasmids were then demethylated by subcloning into *dam*-/*dcm*-competent *E. coli* for further transformation into *H. volcanii*. 

All *ftsZ* and *cetZ* deletions were performed in H53 *H. volcanii* using the polyethylene glycol transformation (PEG) procedure [22] and the “pop-in, pop-out” method [20]. Cells were plated on Hv-CA supplemented with tryptophan for 5–7 days for selection. Colonies were screened for pop-ins by PCR, and those confirmed were plated on Hv-CA plates with 5-flouroorotic acid (5-FOA), uracil, and tryptophan. Successful pop-outs resulting in the desired deletion were confirmed by PCR; we developed forward and reverse external primers (see Supplemental Table S1) specific to each gene. Results from PCR were then visualized on an agarose gel. 

The fluorescence plasmid used for cell visualization, pNUFsmRS-GFP, is a derivative of the plasmid pJAM1020 [23] with *pyrE2* (uracil production) and a *fdx* promoter added. Using the plasmid pTA131 as a template, primers with *Nsi*I/*Pci*I restriction sites were used to PCR-amplify *pyrE2* and the *fdx* promoter. pJAM1020 was digested with *Nsi*I/*Pci*I to linearize and remove ~1.5 kb of non-coding DNA. Using the In-Fusion HD Cloning kit (Clontech), *pyrE2*/*fdx* promoter was inserted into the now smaller, linearized pJAM1020 plasmid. The resulting plasmid, now called pNUFsmRS-GFP, was then successfully screened for GFP and uracil production. Successful deletion mutants were then transformed with the plasmid pNUFsmRS-GFP using the PEG transformation procedure to allow for fluorescent microscopy. 

### 2.3. Liquid Biofilm Formation, Fluorescent Microscopy Imaging, and Analysis 

Biofilms were either grown in 8-well chamber slides (Lab-Tek, #155411) or 35 mm glass-bottom dishes (MatTek, #P35G-1.5-20-C). Logarithmically growing *H. volcanii* cells were diluted to 0.1 OD_600_ in fresh medium with appropriate supplements. For plating, 400 µL of diluted culture was added to each slide chamber, or 4–5 mL of diluted culture was plated in the 35 mm dishes. After inoculation, the cultures were incubated at 42 °C for 1 h, and then microscope imaging was performed. 

All images were taken on an inverted Olympus IX83 FV1200 fluorescent microscope using 488 nm excitation, with a 60× immersion silicone lens (UPLSAPO 60XS, NA 1.30) and a stage-top incubator (Tokai Hit, Fujinomiya, Japan, IX3WX-STX). Both the lens and incubator were heated to 42 °C. Images were taken at the 1 h, 3 h, and 6 h timepoints post plating via the Olympus Fluoview software (FV10-ASW 4.2). Composite Z-stack figures are composed of 0.25 µm/slice in the Z-plane. 

Using FIJI (v1) [24], cell length and roundness (4 × area/(π × major_axis^2^)) measurements were taken. Measurements were taken from 6–7 images per timepoint per replicate, with each deletion having 3 independent biological replicates. Cells were selected via color threshold with a size exclusion of anything below 0.25 µm^2^ and 0.01 circularity. Length measurements were acquired using the measurement function in FIJI with n = 1000 per timepoint (Supplementary File S1). For roundness, the “analyze particles” function was used across three replicates; >2800 measurements were taken for each strain at 1 h, >6000 at 3 h, and >8500 at 6 h (Supplementary File S1). For values closer to one, cells exhibit more roundness, while those closer to zero indicate more elongated cells. A value of 0 denotes a one-dimensional line; however, no cell can have a value of 0 as they all have a width associated with them. A two-sample *t*-test was performed to compare the deletion strains to the parental at each timepoint. To account for any biases, we used a random number generator in Microsoft Excel (Version 2308 Build 16.0.16731.20182) to randomly subsample the length measurements for the parental: *ΔftsZ1*, *ΔcetZ2*, and *ΔcetZ4-6* at n = 250, n = 500, and n = 750, respectively. The deletions were then compared to the parental at the corresponding timepoints and randomized subsampling. 

## 3. Results

### 3.1. Haloferax volcanii Exhibits Three Main Morphologies during Early Liquid Biofilm Formation

**Figure 1:**
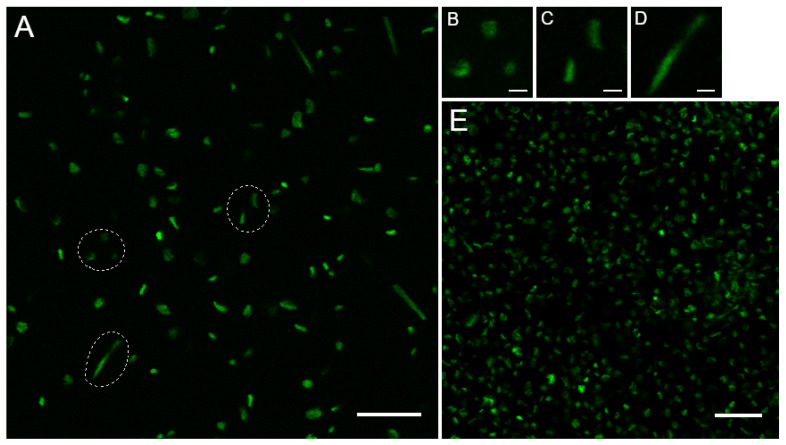
*Haloferax volcanii* exhibits three main morphologies during early liquid biofilm formation. (**A**) Mid-log GFP-expressing H53 (parental) cultures were grown statically in glass-bottom dishes (3 h post plating pictured, scale 15 µm). Three common cell morphologies are highlighted by white circles and shown in the insets: (**B**) discoid, (**C**) rod, and (**D**) filamentous shape, which can be seen in the first three timepoints post plating (scale 2 µm). (**E**) By the 24 h mark, aggregation of cells makes single-cell analysis difficult (scale 15 µm).

To understand the role of the FtsZ and CetZ proteins in controlling cell shape, it was first necessary to create a standardized protocol for biofilm growth, establish experimental timepoints, and characterize the morphologies seen in the *H. volcanii* H53 parental strain when allowed to grow without shaking (i.e., statically). A GFP-expressing H53 strain (pNUFsmRS-GFP) was plated at 0.1 OD_600_ in a round glass-bottom dish to allow biofilm formation and then imaged over the course of 24 h. Within the first hour, not only did cells start to adhere to the dish, but all three morphologies (discoid, rod, and filamentous) could be seen concurrently (Figure 1B–D). By 24 h post plating, there were enough cells attached to the dish that cell aggregation interfered with imaging (Figure 1E). Further analysis focused on the timepoints 1 h, 3 h, and 6 h post plating as they had the best cell densities for imaging and analysis. Later timepoints were not chosen as the continuing formation of larger structures and increased cell confluence made single-cell analysis difficult. In addition, a previous study showed that, under similar conditions, differential gene expression of the tubulin-like family begins to decrease after four hours post initiation [18].

### 3.2. Deletion of ftsZ-cetZ Family Genes Causes Morphological Changes during Biofilm Development

**Figure 2:**
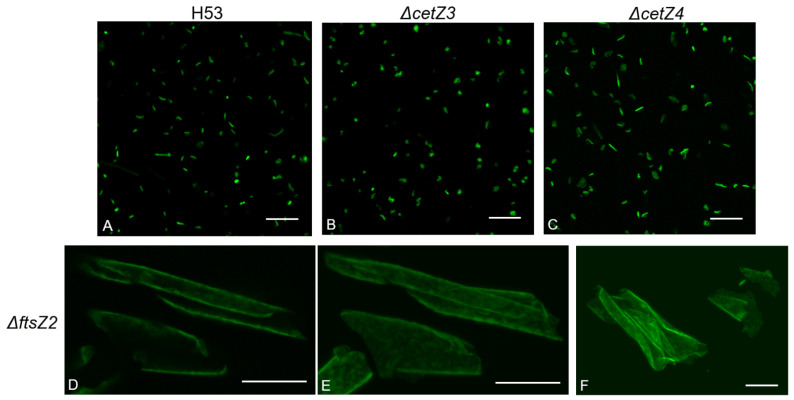
Deletion of *ftsZ*-*cetZ* family genes causes morphological changes during biofilm development. (**B**–**F**) Confocal microscopy visualization showed morphological changes in the deletions when compared to the parental strain (**A**). (**D**–**F**) To better visualize the 3D nature of the *ΔftsZ2* strain, multiple photos in the Z-plane were taken (**D**) to make single composite images (**E**,**F**). All scale bars are 15 µm. See Supplemental Figure S1 for all deletions.

GFP-expressing strains of H53 with single-gene deletions of *ftsZ1-2* and *cetZ1-6* were plated in glass-bottom wells or dishes at 0.1 OD_600_ and imaged at the 1 h, 3 h, and 6 h timepoints post inoculation (Figure S1). Surprisingly, when compared to the parental strain, there were several visual changes in these deletions (Figure 2). Most deletions created an increase in cell length and/or the ability to make filamentous cells; however, two others caused a decrease in cell length and the ability to make rod or filamentous cells. *ΔcetZ1* produced little to no rod morphologies at any timepoint (Figure S1) when grown as a biofilm. Interestingly, *ΔcetZ3* cells seemed similar to *ΔcetZ1* in visual observation (Figure 2B; see statistical analyses below). The visual observations of *ΔcetZ4* (Figure 2C), *ΔcetZ2*, *ΔcetZ5*, *ΔcetZ6*, and *ΔftsZ1* (Figure S1) suggested that they all produced longer cells with more rod and filamentous shapes present compared to the parental strain. Finally, large, 3D plate-like cells that varied in size were observed when *ftsZ2* was deleted (Figure 2D–F). Further, this deletion created none of the standard morphologies seen in the other deletions or the parental strain. The sizes of these plate-like cells vary from one another (mostly longer than 20 µm but can reach over 50 µm at their longest point), and there is no consensus shape other than their flatness, which is a common feature among haloarchaea (e.g., *Haloquadratum walsbyi*) [25]. Despite this, the cells are viable and grow to high density, which contrasts with the requirement of FtsZ in bacterial cells. Due to their unique morphology, *ΔftsZ2* was omitted from further analysis and comparisons in this study. 

### 3.3. Mutant Cells Were Typically Longer Than Parental

**Figure 3:**
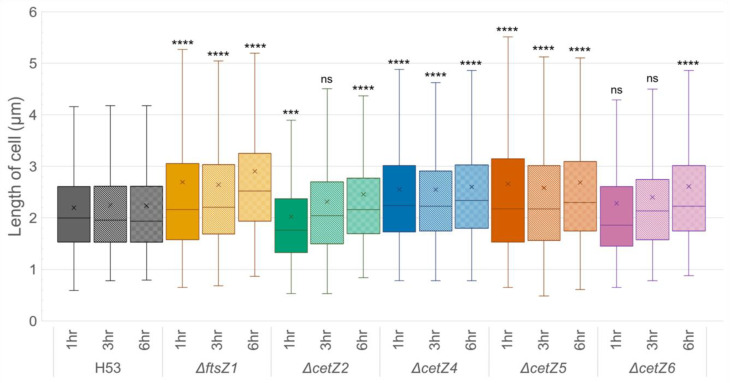
Mutant cells are typically longer than parental cells. Cell length measurements were taken at 1 h, 3 h, and 6 h and compared to the parental strain (H53) at the corresponding timepoints. Box-and-whisker plots are shown; outliers have been hidden. Boxes represent the interquartile, whiskers show upper and lower values, the middle line denotes the median, and “x” represents the mean value. Each timepoint n = 1000. (ns = no significance, *** = *p* < 0.001, **** = *p* < 0.0001.) See Figure 2 and Figure S1 for representative images, and Figure S2 for data represented as SuperPlots.

To determine if the dimensions of deletion mutant cells were statistically different compared to the parental cell line, those that were able to create rod and filamentous shapes were analyzed for differences in length using scientific image processing and analysis software (FIJI, v1) [24] (see Section 2). To account for any randomness in cell orientation that would make rod cells appear shorter than they were, we measured 1000 cell lengths per timepoint per deletion across three independent experimental replicates. Further, we randomly subsampled n = 250, n = 500, and n =750 cells (Table S2). The comparison between cell length averages and the parental shows a statistically significant difference in cell length across all deletions and nearly all timepoints (Figure 3, Table S2). Of the five deletion mutants, *ΔftsZ1*, *ΔcetZ4*, and *ΔcetZ5* produced cells that were all significantly longer (*p* < 0.0001) than the parental at all timepoints measured (1 h, 3 h, and 6 h), with the exception of *ΔcetZ5* at 3 h n = 250, which showed no significant difference to the wild type (Table S2). *ΔcetZ2* cells started significantly shorter (*p* < 0.001) than the parental, but by 6 h, they became significantly longer. Finally, *ΔcetZ6* at the 1 h and 3 h timepoints had cells that were statistically indistinguishable in length from the parental (though there was support at n = 1000 3 h, it was not statistically supported in the random subsampling (Table S2)), but grew to be significantly longer by 6 h. There did not seem to be any impact on the length these mutants could grow, as all strains had cells that grew over 10 µm with maximum cell lengths reaching 20 µm during the six-hour observation time period. 

**Figure 4:**
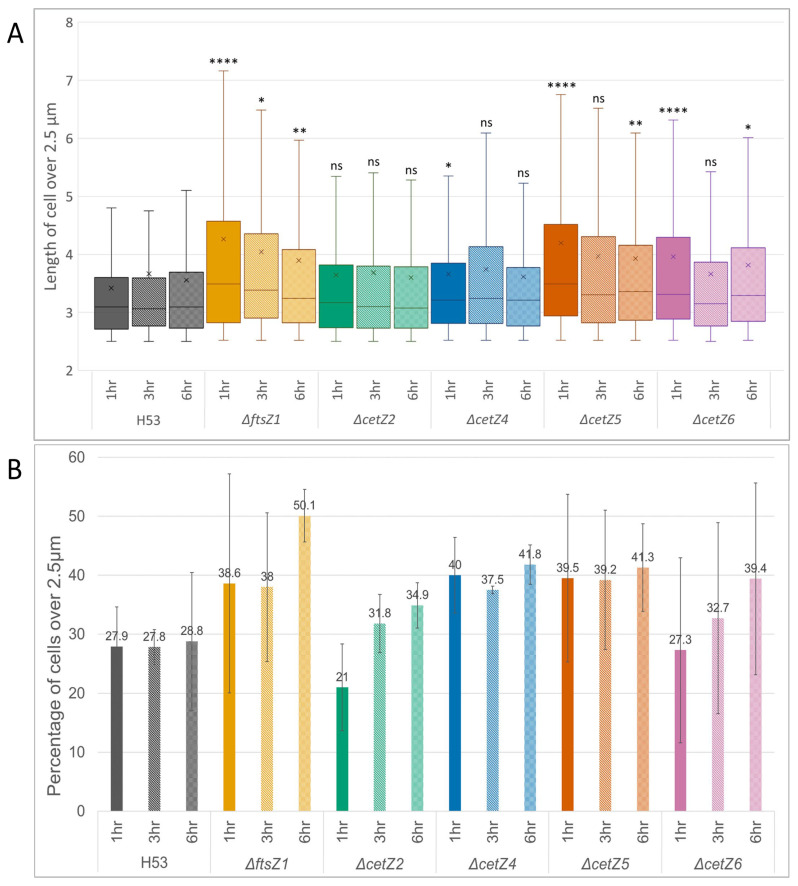
Filamentous cell length and percentage. (**A**) All cell lengths over the 2.5 µm threshold were compared to the parental strain (H53) at the corresponding timepoints, with each timepoint having > 200 cells. (ns = no significance, * = *p* < 0.05, ** = *p* < 0.01, **** = *p* < 0.0001.) (**B**) The distribution of filamentous cells is shown as a percentage. Bars represent SD. See Figure 2 and Figure S1 for representative images.

Using a cutoff of 2.5 µm and above to exclude all smaller discoid and traditional rod-shaped cells from the analysis, the filamentous population of cells was evaluated (Figure 4A). Previous work showed that the average diameter of the discoid shape is not larger than 1.75 µm, and most rod-shape cells fall under 2.5 µm in length [15]. The observations of *ΔftsZ1*, *ΔcetZ5*, and *ΔcetZ6* showed a trend of cells above the 2.5 µm threshold starting off significantly longer than the parental cells at the 1 h mark (*p* < 0.0001), but they decreased in size and significance by the 6 h timepoint. *ΔcetZ2* cells showed no significant difference in length at any of the timepoints compared to the parental cells, whereas *ΔcetZ4* cells started off longer but after 1 h adopted a more parental-like phenotype. Comparing the cells above 2.5 µm to all measurements showed that in the parental strain, filamentous cells made up an average of 28.2% of the population across all timepoints (Figure 4B). By the 6 h mark, each of the deletion mutant strains had a higher percentage of cells over 2.5 µm compared to the parental. *ΔcetZ*4 and *ΔcetZ5* deletions had an average of 37.5% and 39.2% of their cells greater than 2.5 µm in length at every timepoint, respectively. *ΔftsZ1* at 1 h and 3 h had similar percentages to *ΔcetZ*4 and *ΔcetZ5*, but by the 6 h mark, *ΔftsZ1* had the highest population of these filamentous-like cells at 50.1%. *ΔcetZ6* deletion started with a lower percentage of cells over 2.5 µm than the parental strain, but by the 6 h mark, it was comparable to the other mutants. Finally, *ΔcetZ2* deletion started off with 21.0% of its population over the threshold, which was less than the parental, but by the 3 h and 6 h marks, it increased to an average of 36.0% of their cells over 2.5 µm in length.

### 3.4. CetZ1 and CetZ3 Deletions Create More Coccoid or Discoid Morphologies

**Figure 5:**
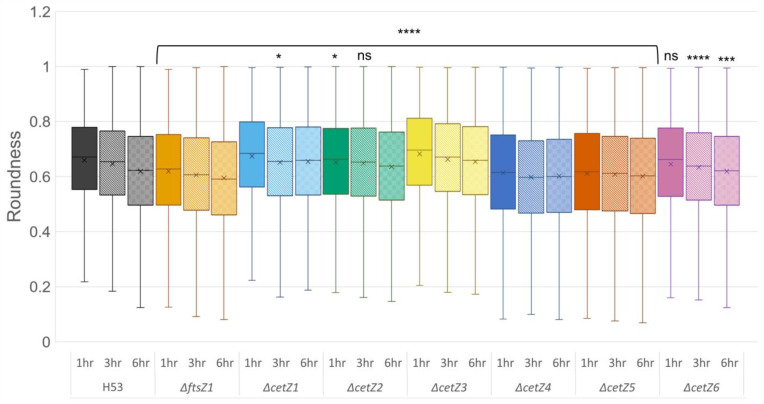
Deletions of *ftsZ1* and all *cetZ* genes have a significant impact on cell roundness. Cell roundness comparisons of parental (H53) and mutants at the corresponding timepoints. A value of 1 is a perfectly round cell, and a value of 0 denotes a one-dimensional line. N = > 2800 at 1 h, >6000 at 3 h, and >8500 at 6 h for each strain. See Figure S3 for data represented as SuperPlots. (ns = no significance, * = *p* < 0.05, *** = *p* < 0.001, **** = *p* < 0.0001.)

To compare all seven deletions in a single analysis, FIJI was used to automate cell roundness measurements (Figure 5). The comparisons of the roundness analyses showed that *ΔftsZ1*, *ΔcetZ4*, and *ΔcetZ5* cells were significantly less round than the parental strain at all timepoints (*p* < 0.0001). At the 1 h timepoint, *ΔcetZ6* cell roundness was not significantly different than the parental, whereas *ΔcetZ2* deletions were rounder, but by the 6 h timepoint, both *∆cetZ2* and *∆cetZ6* became less round than the parental. These results support both the length measurements (Figure 3) and distribution (Figure 5) previously seen, as all the deletions that were significantly longer than the parental were also significantly less round in comparison. The mutant strains *ΔcetZ1* and *ΔcetZ3* had significantly rounder cells than the parental across all the timepoints observed. This confirms that both deletions create little to no rods and are mainly coccoid- or discoid-shaped. 

**Figure 6:**
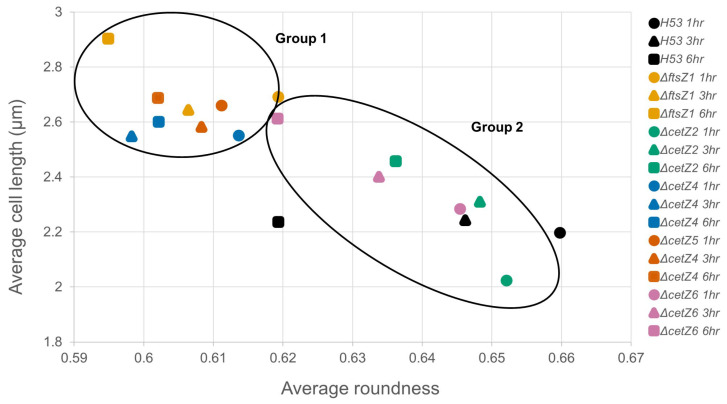
Comparisons of length and roundness measurements. Plotting length and roundness measurements demonstrate two populations, Group 1 and Group 2.

The analysis comparing cell length to roundness measurements (Figure 6) showed the expected inverse relationship, where an increase in cell length caused a decrease in roundness. Visualizing this relationship helped show two distinct populations: a group of *ΔftsZ1*, *ΔcetZ4*, and *ΔcetZ5* deletions that start and end both longer and less round than a second group, which consists of *ΔcetZ2* and *ΔcetZ6*. By 6 h, Group 2 cells were still shorter and rounder in comparison to where Group 1 started at 1 h. Although *ΔcetZ2* and *ΔcetZ6* initially appeared to group with the parental, their cells became significantly longer and less round by 6 h. 

## 4. Discussion

The study of archaea provides opportunities to fill the large knowledge gap in regard to the emergence and evolution of eukaryotes from prokaryotes. Archaea have a mosaic of bacterial and eukaryotic-like processes that remain understudied. The FtsZ/CetZ tubulin-like protein family is one example of this missing link. Archaeal FtsZ is not the result of lateral gene transfer but is believed to have been present in the last common archaeal ancestor [26]. Although CetZ proteins are thought to have evolved from archaeal FtsZ [26], key regions like the GTP-binding domain have greater similarity to eukaryotic tubulin [6], suggesting CetZ as an intermediate form between FtsZ and tubulin. The only archaeal gene that is thought to be more closely related to tubulin than the FtsZ/CetZ family encodes the hypothetical protein artubulin (found in two Thaumarchaeota genome studies) [6,27]. 

Previous analysis showed that FtsZ1/2 and CetZ1 can influence cell shape under standard growth conditions (i.e., shaking liquid cultures), with FtsZs involved in Z-ring formation and CetZ1 involved in creating the rod morphology [7,9]. These phenotypes were maintained when grown as biofilms. However, the remaining *cetZ* gene deletions also indicated different morphologies when cells were observed during early biofilm development conditions.

Since *cetZ1* deletion prevents rod morphology and that there is a filamentous morphology only observed under biofilm conditions, we hypothesized that the other CetZs could be involved in shape determination when cells are grown as biofilms. Differences in cell shape (Figure 3, Figure 4A and Figure 5, and Supplemental Figure S1) were observed and statistically supported for strains in which the deletions of these tubulin-like genes were made. The morphology of *ΔftsZ2* under biofilm growth conditions was similar to the results of *ΔftsZ2* visualized under standard laboratory conditions seen by Liao et al. [9]. The deletions of *cetZ2* and *cetZ4-6* showed a trend of increasing cell length over time and a concomitant decrease in roundness in comparison to the parental. Interestingly, during the first hour, *ΔcetZ2* was the only deletion that was significantly shorter (*p* < 0.001) than the parental cells. In congruence with Duggin et al. [7], deletion of *cetZ1* continued to produce no rods or filaments even when grown in a biofilm; additionally, deletion of *cetZ3* also produced a similar phenotype, with both deletions resulting in cells that were statistically rounder than the parental. This suggests that CetZ4, CetZ5, and CetZ6 have similar functions in maintaining cell shape; CetZ1 and CetZ3 both play roles in rod formation; and CetZ2 might have a unique function among CetZs. CetZ2 might be involved in early cell shape determination in the initiation of biofilms, as its deletion had a negative impact on cell length and filament production in the first hour, but at subsequent timepoints, it appeared similar to the *cetZ4-6* deletions. Although the resulting phenotypes for many cells are similar, the deletions show differences amongst each other in cell length and the percentage of cells over 2.5 µm when compared to the parental. 

The differentiation of cells into a specialized form is not unique to eukaryotes; the Gram-positive model organism *Bacillus subtilis* uses quorum sensing and other chemical signals to differentiate into several subpopulations when grown as a biofilm, with as many as five distinct cell types, each with their own purpose (e.g., motility, sporulation, matrix production) [28,29]. Cell differentiation is not limited to Gram-positive bacteria either, as some cyanobacteria (e.g., genus *Anabaena*) differentiate into cells that undergo photosynthesis or form heterocysts that are able to fix atmospheric nitrogen [30]. Both current and previous work [15] show three distinct cell types for *Haloferax volcanii*: discoid, rod, and filamentous (Figure 1). It is unknown what specialization the discoid shape provides. However, we point out that under high salt concentrations, nutrient diffusion is limited, and having a lower volume-to-surface ratio helps aid in nutrient acquisition and waste dispersal [31]. Under biofilm growth conditions, the development of rods, which are associated with movement [7], can be considered a transitional form as they develop during shaking conditions and proliferate under non-shaking conditions. Filamentous morphology only develops under static biofilm culture conditions [15] and is different from rod morphology, as cells are much longer, thinner, and taper at the ends. We speculate that filamentous cells develop from rod morphologies and are associated with attachment and biofilm matrix production. Filamentous cell growth (cells greater than 2.5 μm) appears tied to both the *ftsZ* and *cetZ* gene families, as five of the eight deletions in these families created an increase in the formation of filamentous cells compared to the parental. These results were only observable when the cells were able to attach and grow on a substratum, which is *H. volcanii*’s presumed state in their natural habitat, as it was originally isolated from Dead Sea sediment [32]. This highlights the difference in phenotype between cells grown in shaking cultures and as a biofilm, which is too often overlooked. We further conjecture that these morphological changes might be controlled by quorum sensing. Although a quorum sensing mechanism in *H. volcanii* has yet to be described, acyl-homoserine lactone-like molecules have been reported in this species [33,34]. Alternatively, they have other environmental sensing mechanisms [35] that could be involved in morphological changes. 

Initial studies regarding the functions of the six CetZ homologs were speculated to either be redundant or have no function [6,7]. Here, we have shown that each *cetZ* deletion impacts cells grown under biofilm conditions, and therefore we suggest they have unique and separate functions. Additionally, multiple studies have shown differences in function between CetZs. A study involving LonB [19] showed that FtsZ1/2, CetZ1, and CetZ5 co-precipitated with LonB, whereas CetZ2-4 and CetZ6 did not. Makkay et al. [18] showed that the FtsZ-CetZ protein family has differing transcription levels under biofilm conditions. Although differing in expression, both *cetZ1* and *cetZ3* produced the same phenotype when deleted, emphasizing the differences among CetZs. The distribution and number of CetZ homologs among the haloarchaea give weight to this proposal, as not every haloarchaeal genus has *cetZ* genes, and many of those that do do not have six (e.g., related *Haloferax mediterranei* has three *cetZ* genes, whereas many others only have one copy) [6,36]. Natural selection is typically strong in large populations, and cells are unlikely to maintain so many copies if they are either functionally redundant or have no function at all. 

We suggest that the plasticity of *Haloferax* membranes may be due to the multiple CetZ homologs and their involvement in cell shape. Like tubulin in the cytoskeleton, these tubulin-like homologs could be acting as support and scaffolding for *H. volcanii*. Multiple studies have shown that a disruption of the tubulin cytoskeleton in eukaryotic cells can lead to changes in cell shape, but in addition, it can interfere with cell differentiation in certain cell types. The differentiation of myoblasts into muscle cells requires the stability of the cytoskeleton to change morphology (elongate) and help cell–cell fusion. When microtubule formation is disrupted, cells can neither elongate nor fuse together to form muscle cells [37,38]. Recent studies showed that disruptions to lipid-/membrane-production proteins (ArtA, HvPssA, and HvPssD) [39], deletion of *sepF* [40], and repression of the transcriptional regulator CdrS [41] all cause changes to cell shape in *H. volcanii*. Since newly made S-layer glycoproteins are added at the midcell during cell growth [39] and FtsZ1/2 co-localizes during division at the midcell [9], it is possible that CetZs are involved in this addition of S-layer glycoproteins to the growing cell. Previous work establishing the localization of CetZ1 shows that there is some localization at the midcell; however, localization is dynamic and not always found at the same place [7]. The FtsZ-CetZ tubulin-like protein family might also be involved in HGT, a process that is intertwined with biofilms and cell shape [15,18]. *H. volcanii* transfers its genetic materials across cytoplasmic bridges during the mating process [16,17,42,43], and it was previously shown that HGT starts rapidly [18]. All this taken together shows the relevance of CetZ proteins to *H. volcanii*’s cell shape and emphasizes the need for more investigation into this family and their roles.

## Data Availability

Data can be found in the Supplementary Materials.

## Supplementary Materials

The following supporting information can be downloaded at https://www.mdpi.com/article/10.3390/genes14101861/s1: Table S1: Description of strains, plasmids, and primers used and their sources; Figure S1: Fluorescent confocal microscopy of *ΔftsZ1* and *ΔcetZ1-6* deletions; Table S2: Significance values of length measurements of *ΔftsZ1*, *ΔcetZ2*, and *ΔcetZ4-6* compared to parental for corresponding randomized subsampling and timepoints; Figure S2: Cell length measurements (Figure 3) represented as SuperPlots; Figure S3: Cell roundness measurements (Figure 5) represented as SuperPlots; Supplementary File S1: Raw cell measurement data.
