# Supplementary material for: Archaeal Tubulin-like Proteins Modify Cell Shape in Haloferax volcanii during Early Biofilm Development
Source: Genes (Basel). 2023 Sep 25;14(10):1861. doi: 10.3390/genes14101861 (PMC10606840; doi:10.3390/genes14101861)
Supplement: Supplementary file 1 [file genes-14-01861-s001.zip › genes-2564306-supplementary.pdf]

Supplementary Materials

Table S1: Description of strains, plasmids, and primers used and their sources; Figure S1: Fluorescent confocal microscopy of *ΔftsZ1* and *ΔcetZ1-6* deletions; Table S2: Significance values of length measurements of *ΔftsZ1*, *ΔcetZ2*, and *ΔcetZ4-6* compared to parental for corresponding randomized subsampling and timepoints; Figure S2: Cell length measurements (Figure 3) represented as SuperPlots; Figure S3: Cell roundness measurements (Figure 5) represented as SuperPlots.

Table S1. Plasmids, strains, and primers.

Plasmid/Strain	Description	Primers used	Primer Sequence (5' to 3')	Source
pTA131	Uracil expressing (*pyrE2*) plasmid used for gene deletion. Allows for selection via blue-white screening (*lacZ*) and ampicillin resistance (Amp^R) in *E. coli.*			Allers et al. [20]
pJAM1020	Plasmid used as basis for pNUFsmRS-GFP construction. Expresses smRS-GFP and allows for selection via Nov^R / Amp^R.			Reuter et al. [23]
pNUFsmRS-GFP	GFP expression plasmid constructed from pJAM1020. Expresses smRS-GFP and allows for selection via NovR / AmpR, as well as *pyrE2* under ferredoxin (*fdx*) promoter.	*pJAM1020_pyrE2_F*	TCC TGT ATG CGA TGC ATC CTT ACT AGA TTA GCC GTC GGC	This study
		pJAM1020_pyrE2_R	GCT GGC CTT TTG CTC ACA CTT TTG ATT TAG ATC CCG TGG ATA A	
H. volcanii H53	Uracil (Δ*pyrE2*) and tryptophan (Δ*trpA*) auxotroph			Allers et al. [20]
Δ*ftsZ1* (H53)	Deletion strain of *ftsZ1* (HVO_0717) derived from H53 (Δ*pyrE2*, Δ*trpA*) with the GFP expressing plasmid pNUFsmRS-GFP (*pyrE2*)	*HVO_0717 ExtF*	TAT AGG GCG AAT TGG GGA GCG ACG AAG AGC AGT G	This study
		HVO_0717 IntR	TAA GCT TAC TGC AGT TAT CTT TCC CCC TTG CGT CAG	
		HVO_0717 ExtR	CTG CAG GAA TTC GAT CTG TAA GAC CAC GCG AGA CC	
		HVO_0717 IntF	ACT GCA GTA AGC TTA TTT TCG CTC CGC TCA GGT TC	
Δ*ftsZ2* (H53)	Deletion strain of *ftsZ2* (HVO_0581) derived from H53 (Δ*pyrE2*, Δ*trpA*) with the GFP expressing plasmid pNUFsmRS-GFP (*pyrE2*)	*HVO_0581 ExtF*	CGG GCC CCC CCT CGA CCG CGC ATC GAA CTC GTC GC	This study
		HVO_0581 IntR	TCA TAT GTC TGC AGT TAA CGC CCT GTC CGA CCC GCG	
		HVO_0581 ExtR	TAG AAC TAG TGG ATC GGT GAT GCG CGA GGC CCT CC	
		HVO_0581 IntF	ACT GCA GAC ATA TGA AGT TAC ACC TTT GCC CAG CCG	
Δ*cetZ1* (H53)	Deletion strain of *cetZ1* (HVO_2204) derived from H53 (Δ*pyrE2*, Δ*trpA*) with the GFP expressing plasmid pNUFsmRS-GFP (*pyrE2*)	*HVO_2204 ExtF*	ATC GAT AAG CTT GAT GGT CAC GAG CGC GTC CGC GAC	This study
		HVO_2204 IntR	ATC CGG AAG TTA ACA TAA GCT GGC GCT CGC GCT CGT C	
		HVO_2204 ExtR	TAG AAC TAG TGG ATC GTC GTC AAC CTC GTC GCG CT	
		HVO_2204 IntF	TGT TAA CTT CCG GAT GTT CAG TCC CCT CGC CAT TGC	
Δ*cetZ2* (H53)	Deletion strain of *cetZ2* (HVO_0745) derived from H53 (Δ*pyrE2*, Δ*trpA*) with the GFP expressing plasmid pNUFsmRS-GFP (*pyrE2*)	*HVO_0745 ExtF*	GCG AAT TGG GTA CCG CCC ATC CGG TCG AGA TAC GA	This study
		HVO_0745 IntR	CCA TAT GCA CGC GTC ACT TTT TCC GTC TCC CTC CC	
		HVO_0745 ExtR	CGG GCT GCA GGA ATT AAC CGT GAG GTT GGA GAC AC	
		HVO_0745 IntF	GAC GCG TGC ATA TGG CGC CCT TGA ACA TTC CTC AC	
Δ*cetZ3* (H53)	Deletion strain of *cetZ3* (HVO_1113) derived from H53 (Δ*pyrE2*, Δ*trpA*) with the GFP expressing plasmid pNUFsmRS-GFP (*pyrE2*)	*HVO_1113 ExtF*	CGG GCC CCC CCT CGA CAG CCT CGA CAT CAA CCA CC	This study
		HVO_1113 IntR	CAT CGA TCC TCG AGC TGA ATA TGC TGT GGG TAG GGC	
		HVO_1113 ExtR	CGG GCT GCA GGA ATT GTG GAT TTC GGC GTT CGA TG	
		HVO_1113 IntF	GCT CGA GGA TCG ATG GGG AAG GGT CAT AGT GCC G	
Δ*cetZ4* (H53)	Deletion strain of *cetZ4* (HVO_A0035) derived from H53 (Δ*pyrE2*, Δ*trpA*) with the GFP expressing plasmid pNUFsmRS-GFP (*pyrE2*)	*HVO_A0035 ExtF*	CGG GCC CCC CCT CGA GAA TCC TGA GAC GCA ACC ACC	This study
		HVO_A0035 IntR	AGG ATC CAT GAT CAA CAA GGC ACC CAT TCG TAA GC	
		HVO_A0035 ExtR	TAG AAC TAG TGG ATC TCC CCG CGT ATG TAC GAT TG	
		HVO_A0035 IntF	TTG ATC ATG GAT CCT ATG GGA CCA CCC TAC CTA CC	
Δ*cetZ5* (H53)	Deletion strain of *cetZ5* (HVO_2013) derived from H53 (Δ*pyrE2*, Δ*trpA*) with the GFP expressing plasmid pNUFsmRS-GFP (*pyrE2*)	*HVO_2013 ExtF*	ATC GAT AAG CTT GAT GAT ACG GCG GGA CCT CTT TC	This study
		HVO_2013 IntR	AGG ATC CAT GAT CAA CGA CTC GCT GTT CTG ACC C	
		HVO_2013 ExtR	TGG CGG CCG CTC TAG GAA CAT CTG CCT GGC GTAC T	
		HVO_2013 IntF	TTG ATC ATG GAT CCT TCT GTT AGT TGC CTG CCA CA	
Δ*cetZ6* (H53)	Deletion strain of *cetZ6* (HVO_2068) derived from H53 (Δ*pyrE2*, Δ*trpA*) with the GFP expressing plasmid pNUFsmRS-GFP (*pyrE2*)	*HVO_2068 ExtF*	CGG GCC CCC CCT CGA GGA CGA CCC TTC TTT CCG ACA	This study
		HVO_2068 IntR	AAG GCC TAG CAT GCA CAC GGG GGT CGA TAA ACG TC	
		HVO_2068 ExtR	CTG CAG GAA TTC GAT AGG CGC TCA ACT ACA TCA CC	
		HVO_2068 IntF	TGC ATG CTA GGC CTT TGT CTG CTC TGC GAC GTA CC	
E. coli HST08 (Stellar)	Strain of *E. coli* used for cloning			Clontech, Cat. #636766
E. coli dam⁻/dcm⁻ (K12)	Strain of *E. coli* used to demethylate plasmids			NEB, Cat. #C2925H

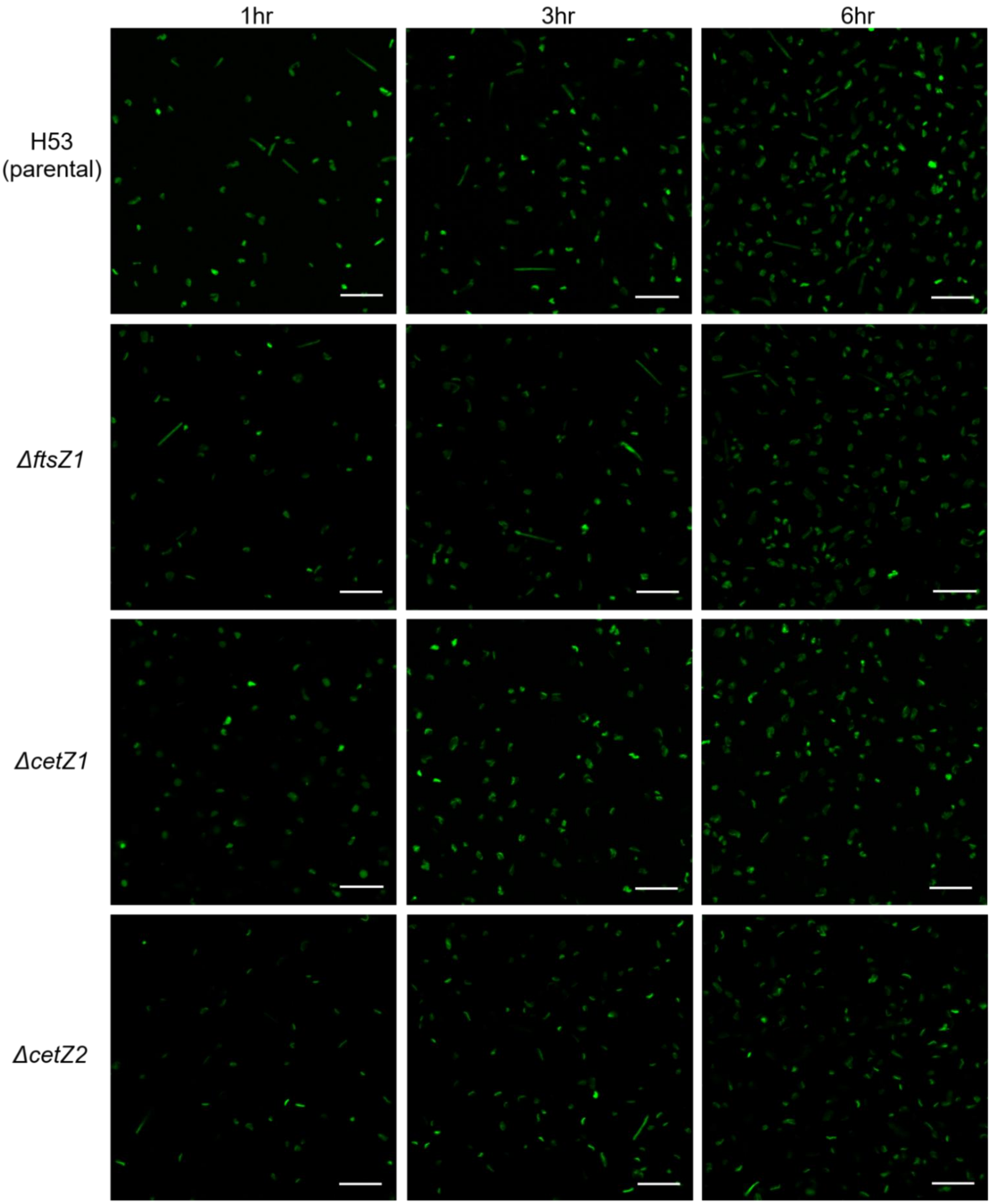
1hr
3hr
6hr
H53 (parental)
ΔftsZ1
ΔcetZ1
ΔcetZ2

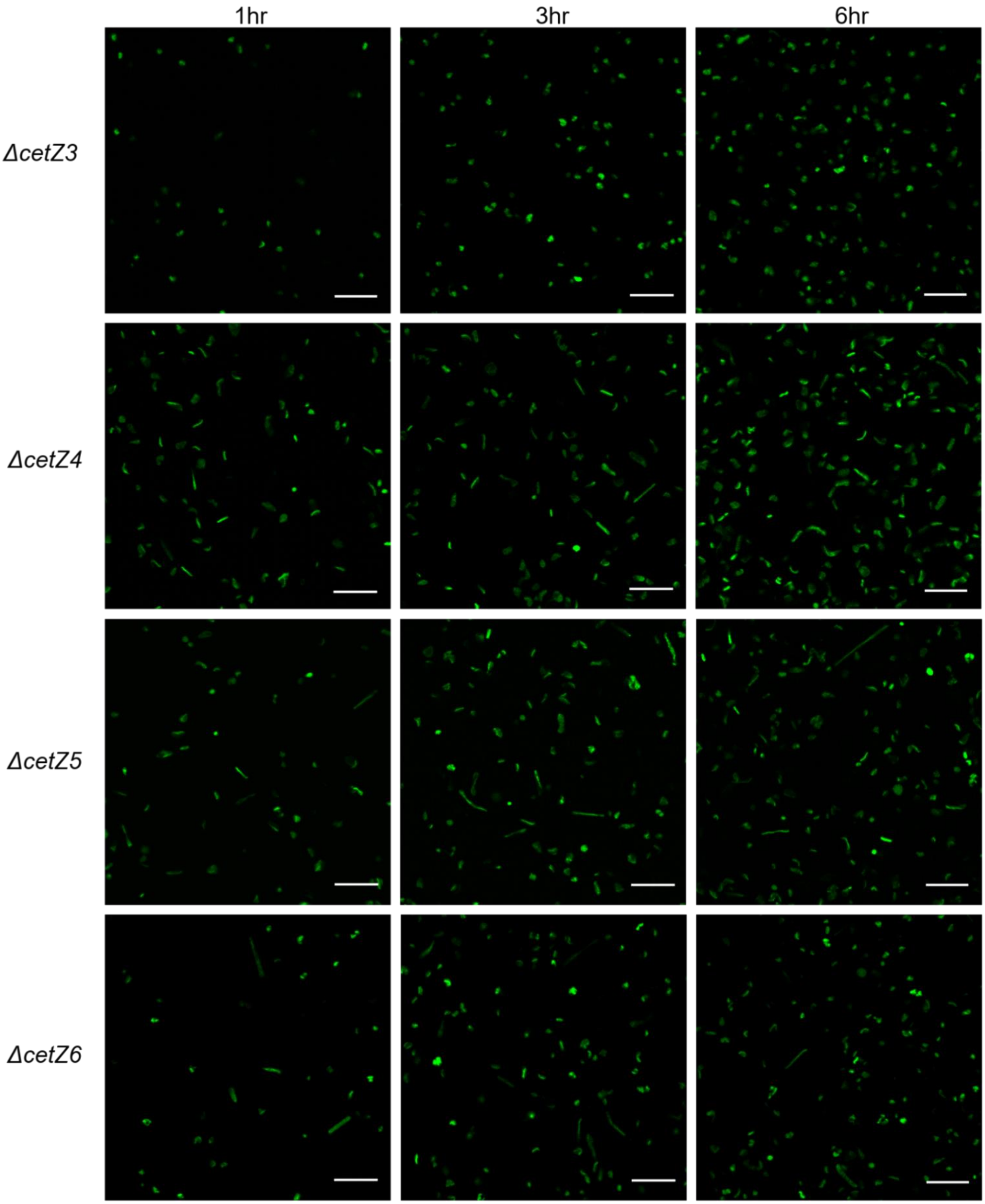

Figure S1: Fluorescent confocal microscopy of *ΔftsZ1* and *ΔcetZ1-6* deletions. Deletions were imaged 1hr, 3hr, and 6hr post plating. Scale bar represents 15 µm.

Table S2. Randomized subsampling: significance values of length measurements when compared to corresponding parental

	ΔftsZ1			*ΔcetZ2*			*ΔcetZ4*			*ΔcetZ5*			*ΔcetZ6*		
	1 hr	3 hr	6 hr	1 hr	3 hr	6 hr	1 hr	3 hr	6 hr	1 hr	3 hr	6 hr	1 hr	3 hr	6 hr
n = 250	*	****	****	**	ns	*	*	**	**	**	ns	****	ns	ns	***
n = 500	****	****	****	*	ns	*	**	****	****	****	****	****	ns	ns	***
n = 750	****	****	****	*	ns	***	****	****	****	****	****	****	ns	ns	****

(ns = no significance, *= p<.05, **= p<.01, *** =p<.001, ****= p<.0001)

= Significantly longer
= Significantly shorter

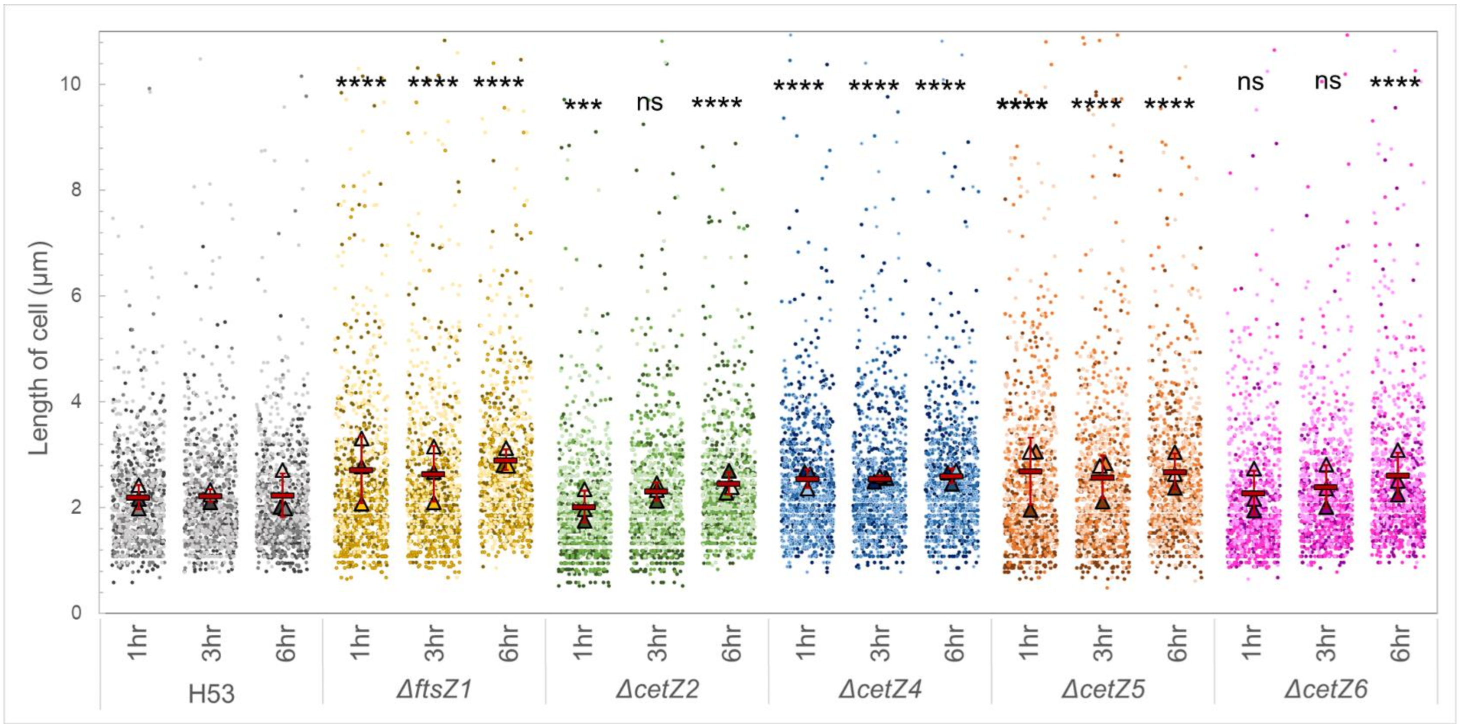

Figure S2. SuperPlots of cell length measurements. Measurements were taken at 1hr, 3hr, and 6hr and compared to the parental strain (H53) at the corresponding timepoints. Red horizontal line represents the mean value with SD shown. Outliers have been hidden. Each timepoint n = 1000. (ns = no significance, *** =p<.001, ****= p<.0001).

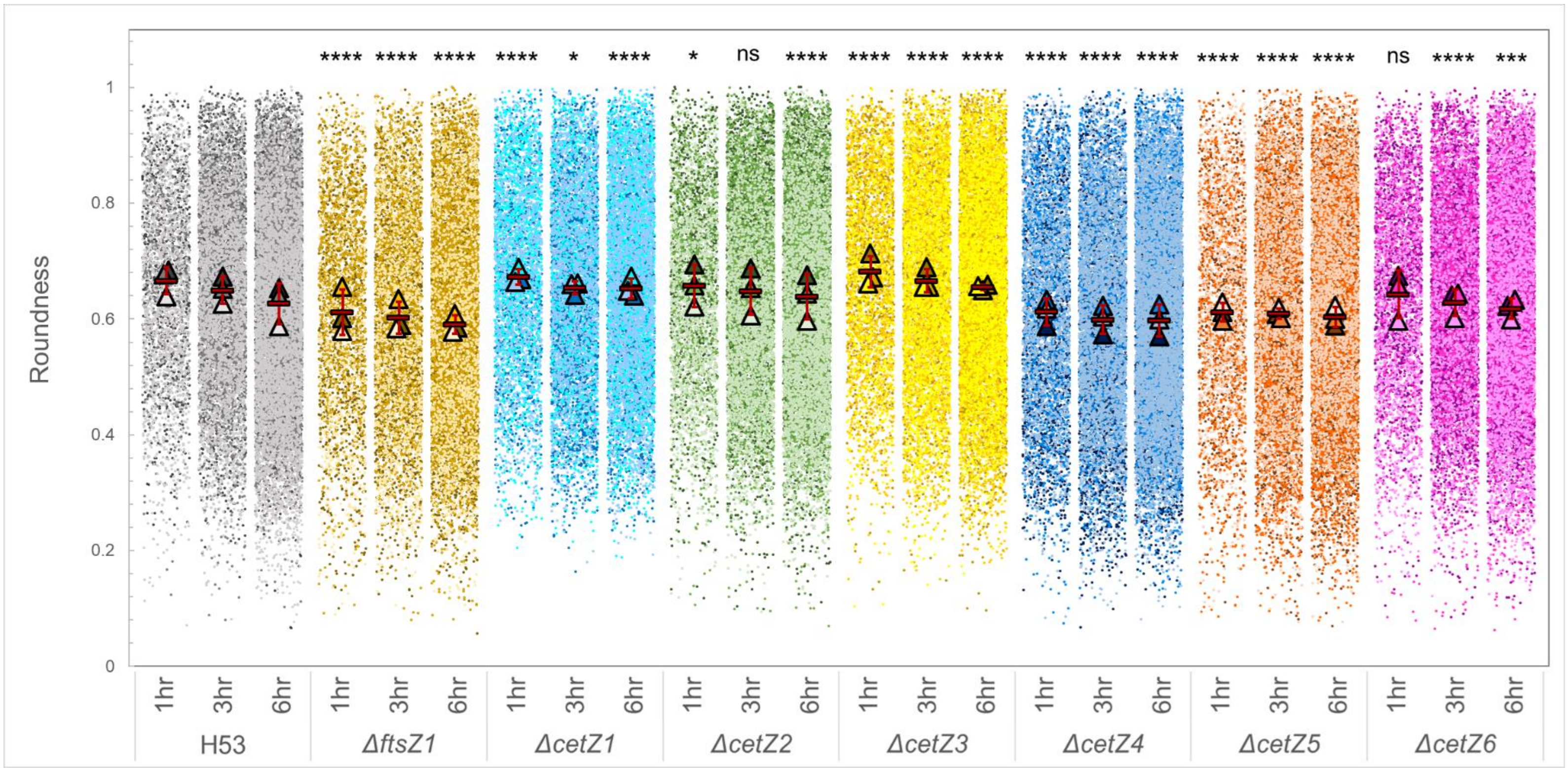

Figure S3. SuperPlots of cell roundness measurements. Cell roundness measurements were taken at 1hr, 3hr, and 6hr and compared to the parental strain (H53) at the corresponding timepoints. Red horizontal line represents the mean value with SD shown. N = > 2,800 at 1hr, > 6,000 at 3hr, and > 8,500 at 6hrs for each strain. (ns = no significance, *= p<.05, **= p<.01, *** =p<.001, ****= p<.0001).
